# Therapeutic benefits of CD90‐negative cardiac stromal cells in rats with a 30‐day chronic infarct

**DOI:** 10.1111/jcmm.13517

**Published:** 2018-01-17

**Authors:** Deliang Shen, Miaoda Shen, Hongxia Liang, Junnan Tang, Bo Wang, Chuang Liu, Peiwen Wang, Jianzeng Dong, Ling Li, Jinying Zhang, Thomas G. Caranasos

**Affiliations:** ^1^ Department of Cardiology the First Affiliated Hospital of Zhengzhou University Zhengzhou Henan Province China; ^2^ Department of Orthopedic Surgery the First Affiliated Hospital College of Medicine Zhejiang University Zhejiang China; ^3^ Department of Infectious Diseases the First Affiliated Hospital of Zhengzhou University Zhengzhou Henan Province China; ^4^ Department of Cardiology Beijing Anzhen Hospital of Capital Medical University Beijing China; ^5^ Division of Cardiothoracic Surgery University of North Carolina at Chapel Hill Chapel Hill NC USA

**Keywords:** cardiac regeneration, myocardial infarction, stem cells, CD90

## Abstract

Cardiac stromal cells (CSCs) can be derived from explant cultures, and a subgroup of these cells is viewed as cardiac mesenchymal stem cells due to their expression of CD90. Here, we sought to determine the therapeutic potential of CD90‐positive and CD90‐negative CSCs in a rat model of chronic myocardial infarction. We obtain CD90‐positive and CD90‐negative fractions of CSCs from rat myocardial tissue explant cultures by magnetically activated cell sorting. *In vitro*, CD90‐negative CSCs outperform CD90‐positive CSCs in tube formation and cardiomyocyte functional assays. In rats with a 30‐day infarct, injection of CD90‐negative CSCs augments cardiac function in the infarct in a way superior to that from CD90‐positive CSCs and unsorted CSCs. Histological analysis revealed that CD90‐negative CSCs increase vascularization in the infarct. Our results suggest that CD90‐negative CSCs could be a development candidate as a new cell therapy product for chronic myocardial infarction.

## Introduction

Cardiovascular diseases remain the number one killer in developed countries 1. Unlike hearts from fishes and amphibians, human hearts have very limited ability for self‐repair 2. Current pharmacological therapies focus on the prevention of secondary damages after acute injury such as myocardial infarction (MI) 3. After MI, viable myocardium is replaced with scarring tissue which will subsequently contribute to advanced cardiomyopathy or heart failure 4. So far, no FDA‐approved therapy can reduce the size of mature scar on the heart. Stem cell therapy and regenerative medicine approaches are aimed to alter the course of maladaptive cardiac remodelling and regenerate damaged heart tissues 5. Resident cardiac stem cells such as c‐kit‐positive cells and cardiosphere‐determined cells (CDCs) have entered early‐phase clinical trials 6, 7. The CDC process starts from culturing cells from myocardial tissue explants 8. These tissue explants generate cardiac stromal cells (CSCs) which form the starting materials that subsequently generate cardiospheres and CDCs. It has been reported that CSCs share similar antigenic identities as CDCs 9. Particularly, CSCs consistently express the TGF‐β receptor subunit CD105 (endoglin); however, they are negative for the haematopoietic markers such as CD45, CD31 and CD34. Moreover, injection of CSCs leads to improvement of cardiac functions and such benefits are similar to those from the injection of CDCs 9. Interestingly, CSCs contain a variable division of CD90 (Thy‐1)‐positive cells. These CD90‐positive cells are commonly viewed as resident cardiac mesenchymal cells. It is elusive which subpopulations of CSCs determine the therapeutic benefits of CSCs. We hypothesize that the CD90‐positive cardiac MSCs are the active principles in CSCs and account for the therapeutic benefits.

## Methods

### Generation of rat CSCs

Rat CSCs were generated and expanded as described from myocardial specimens 10. Rat hearts from S.D. male rats (6–10 weeks) were harvested and minced into 1–2 mm^3^ small tissue explants. Those tissue explants were digested in collagenase (Sigma‐Aldrich, St. Louis, Missouri, USA) for 5 min. and then plated on to fibronectin‐coated dishes with shallow Iscove's Modified Dulbecco's Medium (IMDM), basic medium (Life Tech Inc., Grand Island, NY, USA) supplemented with 10% FBS (Hyclone) and 20 mg/ml gentamicin (to control bacteria growth); 7–10 days after plating the tissue explants, cardiac stromal cells (CSCs) will spontaneously migrate out from the tissue explants. They can be further passaged and expanded with standard cell subculture techniques.

### CSC characterization and sorting

Unsorted CSCs were characterized by flow cytometry as described 11. Cells were incubated with FITC‐, PE‐ or APC‐conjugated antibodies (from R&D Systems or BD Biosciences) against CD105, CD45 and CD90 for 60 min. Isotype‐identical antibodies served as negative controls. Quantitative analysis was performed using a CyAN flow cytometer with FlowJo software (Ashland, OR). Magnetically activated cell sorting (MACS) was performed using anti‐CD90 microbeads (Miltenyi Biotec) according to the manufacture's instructions. Such cell sorting fractionated unsorted CSCs into CD90‐positive CSCs (or CD90+ CSCs) and CD90‐negative CSCs (CD90− CSCs). To enable cell fate tracking, a cohort of cells were genetically labelled with green fluorescent protein (GFP). Ready‐to‐use pre‐made GFP lentiviral particles were purchased from GenTarget Inc (San Diego, USA). Briefly, CSCs were grown to 50–75% confluent in 24‐well plate. The culture media were removed and replaced with fresh and warm culture media. The lentiviral particles were thawed and added to the cell culture media at a MOI of 100:1. The cells were returned to the incubator; 72 hrs after transduction, the GFP positive cells were observed with a fluorescence microscopy.

### Cardiomyocyte assay

Neonatal rat cardiomyocytes (NRCMs) were derived as previously described from SDrats 12. NRCMs were plated onto fibronectin‐coated chamber slides with 2% FBS media. After 3 days, NRCMs were cultured with the IMDM conditioned media from unsorted CSCs, CD90+ CSCs or CD90− CSCs. Cardiomyocyte number and beating were evaluated with white‐light microscopy. ki67 and TUNEL staining were performed to examine the proliferation of apoptosis of cardiomyocytes.

### Tube formation assay

Human umbilical vein endothelial cells (HUVECs) were purchased from ATCC. Before we investigated the pro‐angiogenic potency, 4000 HUVECs were plated onto Matrigel™ (BD)‐coated 96‐well plate with vascular cell basal medium. For tube formation assay, conditioned media from CSCs, CD90+ CSCs or CD90− CSCs were collected and added into the wells; 6 hrs later, tube formation from HUVECs was observed with a white‐light microscope and average tube lengths were measured by ImageJ (NIH).

### Protein array

To compare the production of various growth factors and cytokines, unsorted CSCs, CD90+ CSCs or CD90− CSCs were seeded in 6‐well culture plates at densities of 1 × 10^6^ cells/ml in FBS‐free IMDM media for 3 days. The supernatants (conditioned media) were collected, and the concentrations of VEGF, bFGF and HGF were measured with rat‐specific ELISA kits (R&D Systems Inc. Minneapolis, MN , USA), according to the manufacturer's instructions. Secretion of various inflammatory cytokines was visualized by a semi‐quantitative antibody array (RayBiotech, GA) and the intensity was determined using NIH ImageJ software.

### Rat chronic myocardial infarction model and cell injection

All animal studies were approved by Zhengzhou University Animal Care and Usage Committee guidelines of Zhengzhou University Animal Care and Usage Committee Guidelines. Male S.D. rats (8–10 weeks old) were raised inside plastic cages (two animals per cage) with 23°C controlled temperature, diurnal light–dark cycle (L:D = 12:12) and with rat chow provided *ad libitum*. The animals were randomly assigned to one of four groups: MI control, MI plus injection of unsorted CSCs, MI plus injection of CD90+ CSCs and MI plus injection of CD90− CSCs. MI was created in female S.D. rats by permanent ligation of the left anterior descending artery 11; 30 days after the infarct, a secondary surgery was performed and hearts were injected at four points in the infarct border zone with a total of 100 μl of one of the following interventions: phosphate‐buffered saline (Control, *n* = 8 rats), 1 × 10^6^ unsorted CSCs (*n* = 14 rats), 1 × 10^6^ CD90+ CSCs (*n* = 14 rats) or 1 × 10^6^ CD90− CDCs (*n* = 14 rats). A cohort of animals received GFP‐tagged cells to allow histological detection 13.

### Cardiac function assessment

Transthoracic echocardiography was performed to evaluate LV function with Vivid 7 ultrasound machine with a small animal probe (GE, Fairfield, CT, USA). After the induction of general anaesthesia, the rat hearts were imaged 2D echocardiography in long‐axis views and short‐axis views. The observer was blinded to the group allocation and collected the following data including of the greatest LV diameter. LV end‐diastolic volume, LV end‐systolic volume and LV ejection fraction were determined with Vivid 7 software.

### Heart histology

For heart histology, all animals were killed at 6 months after cell injection (after echocardiography study), and excised hearts were cryosectioned (5 μm thickness). Heart cryosections were then fixed with 4% PFA, blocked/permeabilized and stained with rabbit anti‐GFP (Abcam) and mouse anti‐α sarcomeric actin (Sigma‐Aldrich) antibodies. Images were taken with a confocal microscope. To quantify myocyte signals, the images were split into difference fluorescent channels with the Image J software. The fluorescent intensity of the myocyte (alpha‐SA; red) channel was measured and normalized to the total intensity 14, 15.

### Statistical analysis

Results are presented as mean ± S.D. unless specified otherwise. Comparisons between any two groups were performed using two‐tailed unpaired Student's *t*‐test. Comparisons among more than two groups were performed using ANOVA for multiple comparisons with post hoc Bonferroni correction. Differences were considered statistically significant when *P* < 0.05.

## Results

### CSC characterization and sorting

**Figure 1:**
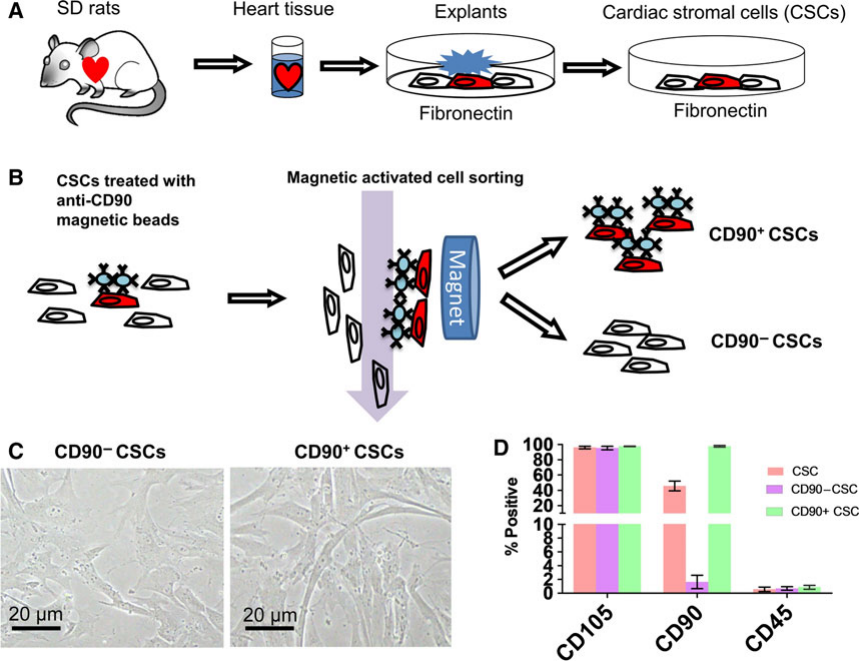
Generation and characterization of CD90‐positive and CD90‐negative CSCs. (**A**) The process of generating rat CSCs. (**B**) cell sorting process. (**C**) The morphology of rat CSCs. (**D**) Flow cytometry analysis of unsorted CSCs, CD90− and CD90+ CSCs (*n* = 3). Scale bar = 20 μm.

The overall study design is outlined in Figure S1. Figure 1A depicts the process of generating rat CSCs, and Figure 1B depicts the process of cell sorting. The morphologies of CD90+ and CD90− CSCs are similar (Fig. 1C). Consistent with previous reports, unsorted CSCs are CD105^High^, CD90^medium^ and CD45^NEG^ (Fig. 1D). After MACS sorting, we enriched CD90− and CD90+ CSCs.

### *In vitro* cardiomyocyte and endothelial cell‐based assays

**Figure 2:**
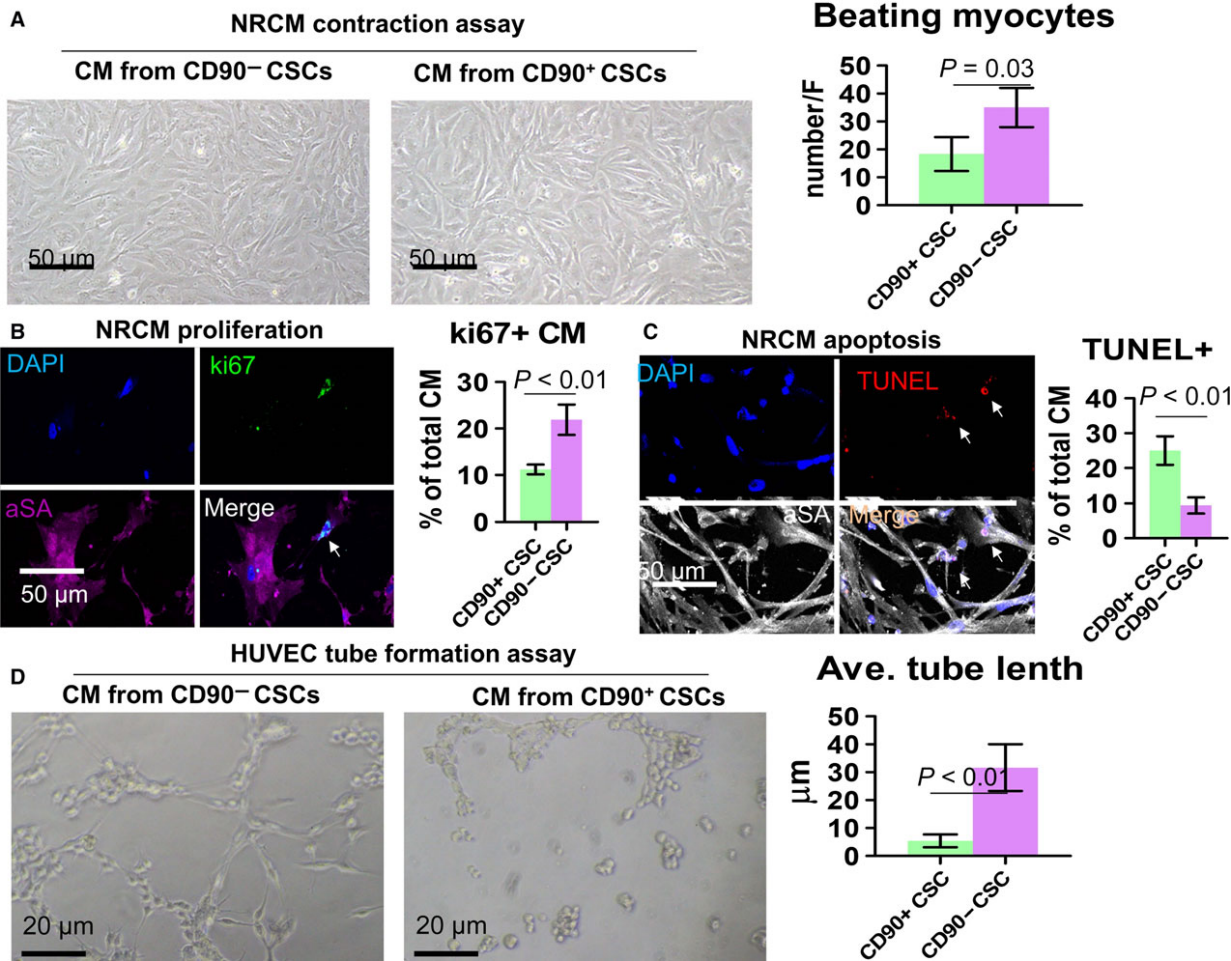
*In vitro* cardiomyocyte and endothelial cell‐based assays. (**A**) NRCMs cocultured with conditioned media from CD90− or CD90+ CSCs. Scale bar = 50 μm. (**B** and **C**) Representative fluorescent micrographs showing ki67 positive (**B**) and TUNEL positive (**C**) cells in NRCM cultures. **D**: HUVEC tube formation on Matrigel in the presence of conditioned media from CD90− or CD90+ CSCs. Scale bar = 20 μm. Two‐tailed *t*‐test for comparison.

When cocultured with NRCMs, conditioned media from CD90− CSCs robustly promote cardiomyocyte survival (Fig. 2A) and contraction (Videos S1–S2), in a fashion superior to that from CD90+ CSCs. Immunocytochemistry revealed that more ki67 positive cells but less TUNEL positive cells in cardiomyocytes cultured with conditioned media from CD90− CSCs (Fig. 2B and C). Tube formation assay indicated higher tube formation level in HUVECs cultured in conditioned media from CD90− CSCs than that from CD90+ CSCs (Fig. 2D).

### Protein secretion assay

**Figure 3:**
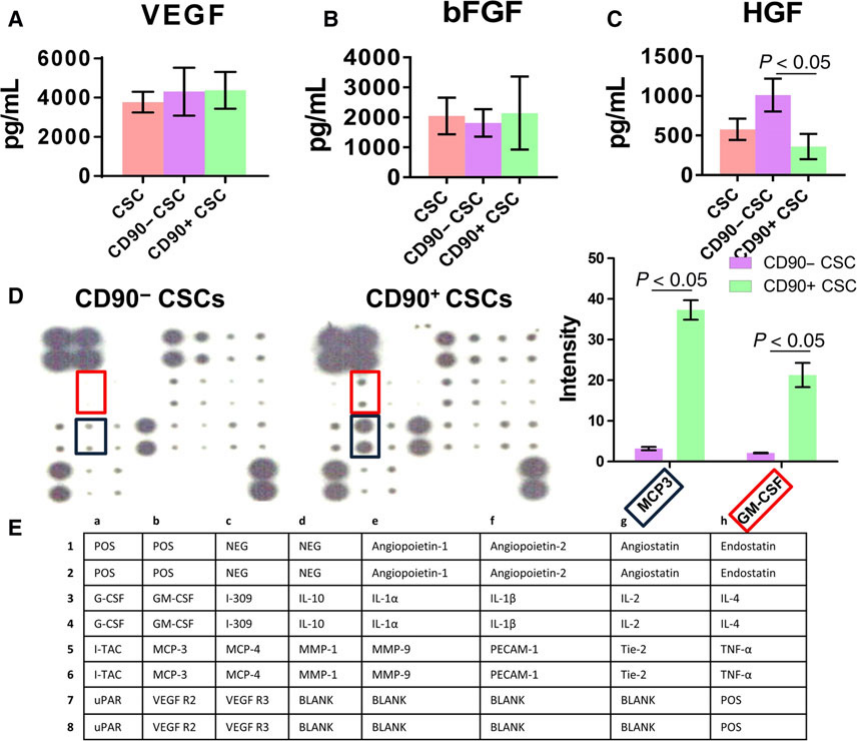
Protein secretion assay. (**A**–**C**) Secretion of various growth factors by CD90− and CD90+ CSCs. Concentrations were measured by enzyme‐linked immunosorbent assay (ELISA). (**D**) Secretion of various inflammatory cytokines by CD90− and CD90+ CSCs. (**E**) Map showing the various proteins measured by the protein array. One way ANOVA with post hoc Bonferroni correction for comparison in (**A**–**C**). Two‐tailed *t*‐test for comparison for comparison in (**D**).

ELISA revealed that CD90− CSCs secreted more HGF than CD90+ CSCs did (Fig. 3C), while the secretion of other paracrine factors such as VEGF and IGF was indistinguishable among the three groups (Fig. 3A and B). Inflammatory cytokine array indicated that less MCP3 and GM‐CSF were secreted by CD90− CSCs (Fig. 3D and E).

### Injection of CD90− CSC promotes cardiac function

**Figure 4:**
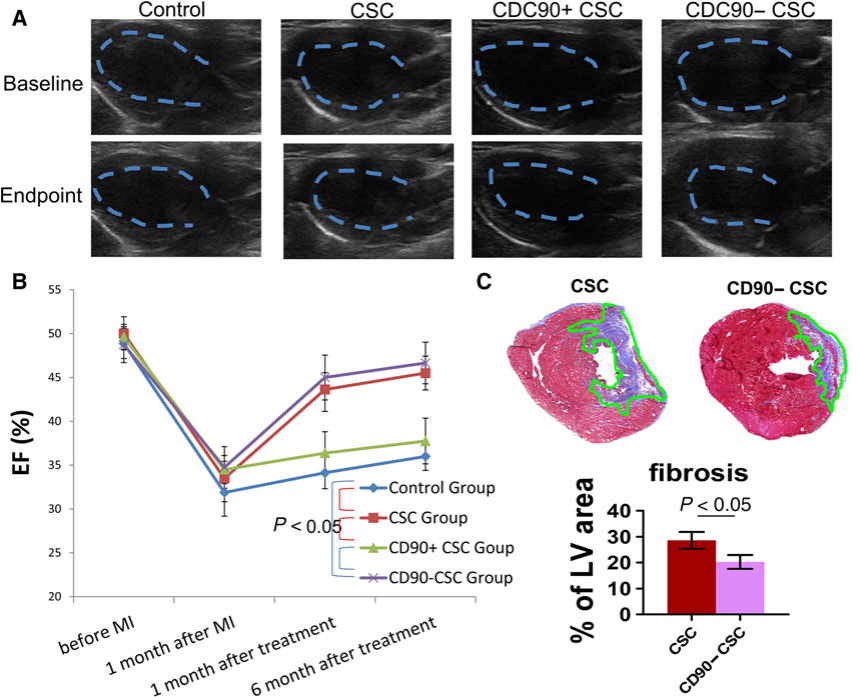
Cardiac function and heart morphometry after cell transplantation. (**A**) Representative echocardiography images showing hearts at baseline and end‐point after various treatments. (**B**) Change in left ventricular ejection fraction (LVEFs). (**C**) Masson's Trichrome staining and measurement of fibrosis area in the heart (*n* = 3). One way ANOVA with post hoc Bonferroni correction for comparison in (**B**). Two‐tailed *t*‐test for comparison for comparison in (**C**).

The bona fide indicator of cell therapy in heart diseases is the ability to protect/augment cardiac function as gauged by echocardiography. We used left ventricular ejection fraction (LVEF) as an indication of cardiac function. Representative echocardiography images were shown in Figure 4A; 30 days after the infarct procedure, all four groups experienced sizable LVEF deterioration from the baseline, indicating a similar degree of cardiac injury, and 6 months after cell injection, the hearts received CD90− CSCs exhibited the highest LVEFs among the groups (Fig. 4B). Unsorted CSCs and CD90− CSCs outperformed CD90+ CSCs, which was indistinguishable with control injections. Masson's Trichrome staining confirmed that CD90− CSCs outperformed unsorted CSCs in reducing fibrosis in the post‐MI heart (Fig. 4C). These compound data sets indicated that CD90− cells are like to be the active therapeutic principles in CSCs.

### CD90− CSC injection promotes cardiomyogenesis in the post‐MI heart

**Figure 5:**
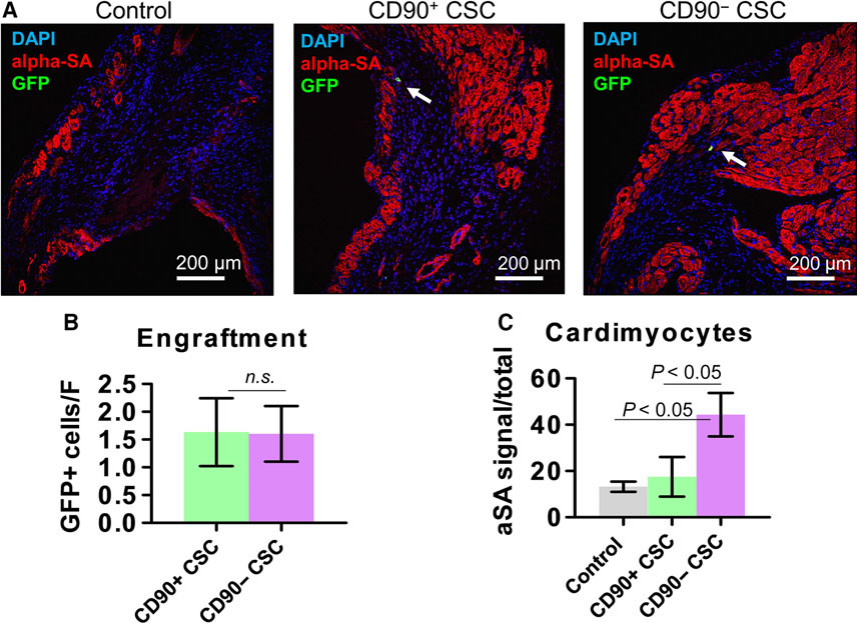
Engraftment and cardiomyogenesis after cell transplantation. (**A**) Representative confocal images showing the engraftment of transplanted CD90− and CD90+ CSCs (GFP‐labelled; green) and cardiomyocytes (alpha‐SA‐labelled; red) in the infarct area. (**B**–**C**) Quantitation of cell engraftment and numbers of cardiomyocytes (*n* = 3). Scale bar = 200 μm. Two‐tailed *t*‐test for comparison.

**Figure 6:**
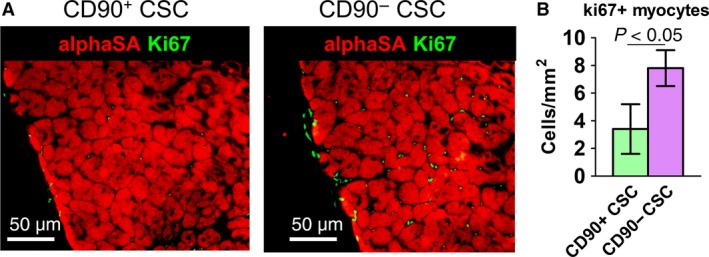
Proliferation of cardiomyocytes. (**A**) Representative fluorescent micrographs showing ki67 positive nuclei (green) in the myocardium. (**B**) Quantitation of ki67 and alpha‐SA double positive cells (*n* = 3). Scale bar = 50 μm. Two‐tailed *t*‐test for comparison.

It has been reported that injected adult stem cells did not persist long‐termly in the heart 16. GFP transduction in CSCs allowed us to track cell fate in the heart; 6 months after cell injection, only negligible numbers of injected cells were still detectable in the heart (Fig. 5A, green). In addition, these cells did not acquire a cardiac phenotype. The numbers of GFP+ cells were indistinguishable between the CD90− CSC and CD90+ CSC groups, suggesting the CD90− cells did not have an engraftment privilege (Fig. 5B). However, more cardiomyocytes were detected in the infarct border zone of animals received CD90− cells (Fig. 5C) than those received CD90+ CSCs. In addition, more ki67+ cardiomyocytes were detected in the hearts treated with CD90− CSCs than those treated with CD90+ CSCs (Fig. 6).

## Discussion

Seventeen years of effort have been dedicated to cardiac cell therapy. Back in the early 2000s, it was reported that transplants of bone marrow cells or c‐Kit+ cardiac cells can regenerate the infarcted rodent heart 17. Later on, the field shifted its paradigm as it became clear that injected cells did not undergo cardiomyocyte differentiation 18. Hypothesis about biological mechanism turned to the activity of putative paracrine secretion by the injected cells 19, 20. Nevertheless, the elusive mechanisms of cardiac cell therapy did not slow down the process of clinical translation. Many cell types have entered clinical stage of investigation. While safety end‐points were generally met, efficacy results have been consistently marginal and moderate. A 2015 review of bone‐marrow‐cell trials for heart attack found no benefits in cardiovascular mortality and cardiac function 21. Cell therapy trials for heart disease still have long pathway to gain FDA approval 22.

Here, we investigated the regenerative potential of cardiac stromal cells derived from myocardium tissue explants. A fraction of those cells was positive for CD90. CD90 (well‐known as Thy‐1) was originally discovered as a thymocyte antigen 23. CD90 is also widely used as a marker of a variety of stem cells, for example MSCs, hepatic stem cells, keratinocyte stem cells, putative endometrial progenitor/stem cells and haematopoietic stem cells 24. In humans, Thy‐1 is also expressed by endothelial cells, smooth muscle cells, a subset of CD34+ bone marrow cells, fibroblasts and foetal liver‐derived haematopoietic cells. Our original hypothesis was the CD90+ CSCs (presumably representing the cardiac MSC population in the heart) play the important role in the therapeutic benefits of CSCs. After testing we found the therapeutic benefits were mainly from the CD90− CSCs (Fig. 4). The CD90− CSCs also outperformed unsorted CSCs in promoting myogenesis (Figs 5 and 6). Therefore, our study leads to a hypothesis disproven that it is not the CD90+ population but is the CD90− population in CSCs mediating the treatment effects in chronic model.

Although the mechanisms underlying this discovery are not completely revealed, our study did provide some hints. *In vitro* cell‐based assays confirmed the superiority of CD90− cells in promoting cardiomyocyte and endothelial cell functions (Fig. 2). This can be further attributed to increased secretion of pro‐angiogeneic factors such as HGF by CD90− CSCs (Fig. 3). In addition, less pro‐inflammatory cytokines were produced by CD90− CSCs (Fig. 3). These data sets suggest the CD90− fraction in CSCs was a pro‐regenerative phenotype while the CD90+ fraction was pro‐inflammatory. Our study also has several limitations. For instance, the key molecules dictating the differences between CD90‐positive and CD90‐negative cells still remain elusive. In addition, as an emerging hot topic of the field, exosomes and microvesicles play important roles in mediating stem cell therapies 25. Future studies on comparing the vesicles from CD90‐positive and CD90‐negative cells are warranted.

In conclusion, our study provided evidence that the CD90− cardiac stromal cells are a previously un‐recognized cell type which confers regenerative property in chronic infarct. Future studies should focus on unveiling the origin and further characterization the phenotypes of this cell population.

## Conflict of interest

The authors indicate no potential conflict of interest.

## Supporting information


**Figure S1** The overall study design.

**Video S1** Cocultured with NRCMs, conditioned media from CD90+CSCs is not good for cardiomyocyte contraction compared with conditioned media from CD90‐CSCs.

**Video S2** Cocultured with NRCMs, conditioned media from CD90‐CSCs robustly promote cardiomyocyte contraction.
