# Supplementary figures and images for: Therapeutic benefits of CD90‐negative cardiac stromal cells in rats with a 30‐day chronic infarct
Source: J Cell Mol Med. 2018 Jan 17;22(3):1984–91. doi: 10.1111/jcmm.13517 (PMC5824400; doi:10.1111/jcmm.13517)

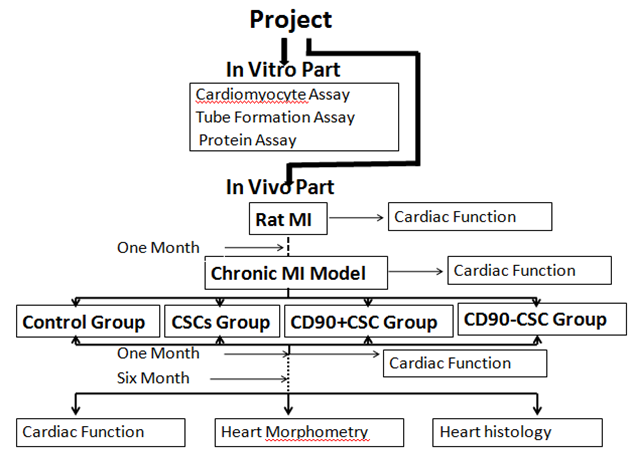

Supplement: Supplementary file 1 — Figure S1 The overall study design. [file JCMM-22-1984-s001.tif]
